# Incidence and predictors of elevated postpartum alanine aminotransferase in chronic hepatitis B mothers: a prospective study protocol

**DOI:** 10.1186/s12876-023-02966-2

**Published:** 2023-11-11

**Authors:** Shi OuYang, Ziren Chen, Tingting Peng, Yawen Geng, Junchao Qiu, Zhirong Xiao, Calvin Q. Pan

**Affiliations:** 1 Department of Infectious Diseases, Key Laboratory of Biological Targeting Diagnosis, Therapy, and Rehabilitation of Guangdong Higher Education Institutes, The Fifth Affiliated Hospital of Guangzhou Medical University, No. 621, Gangwan Road, Huangpu District, Guangzhou, 510799 China; 2 School of Public Health, North China University of Science and Technology, Tangshan, China; 3 Department of Obstetrics, Key Laboratory of Biological Targeting Diagnosis, Therapy, and Rehabilitation of Guangdong Higher Education Institutes, The Fifth Affiliated Hospital of Guangzhou Medical University, Guangzhou, China; 4 Division of Gastroenterology and Hepatology, Department of Medicine, NYU Langone Health, New York University Grossman School of Medicine, New York, USA

**Keywords:** Chronic hepatitis B, Mother-to-child-transmission, ALT flares, Postpartum monitoring, Risk for ALT elevation.

## Abstract

**Background:**

The majority of HBeAg-positive mothers with chronic hepatitis B have high levels of viremia and inactive disease with normal alanine aminotransferase (ALT) during pregnancy. In addition, postpartum disease activation and ALT flare have been reported in the range of 15 − 35%. However, the current International Association Guidelines have not provided clear recommendations and a risk-stratified monitoring schedule. Furthermore, data are lacking on the definition of normal ALT in the postpartum period in mothers with chronic hepatitis B. The clinical features and ALT flare patterns in HBeAg-positive mothers versus HBeAg-negative mothers are not fully explored. Thus, we design a cohort study to investigate the aforementioned area and generate data to assist healthcare providers in better managing mothers with hepatitis B. We aim to assess the frequency of postpartum ALT flares and predictors for such events.

**Method:**

This study is a single-center and prospective cohort study (n = 360) that consists of two groups of patients including HBsAg-positive mothers (n = 120) and healthy mothers without HBV infection (n = 240). In HBeAg-positive mothers, antiviral therapy during late pregnancy is permitted to prevent Mother-to-child transmission (MTCT) but discontinued at delivery if there is no further indication for the treatment. Mothers are enrolled at the gestational weeks of 12–24. After delivery, both mothers and their infants will be followed up until postpartum week 24. Clinical and laboratory data are collected every 4 weeks during the study except there are no follow-up visits at the postpartum weeks 16 and 20. The primary objective is the proportion of patients with postpartum ALT flares. The secondary objectives are independent risk factors during pregnancy for predicting postpartum ALT flares and the normal range of postpartum ALT levels in healthy mothers.

**Discussion:**

The current study focuses on the incidence of postpartum ALT flares in mothers with chronic hepatitis B including subgroup analysis based on HBeAg status. The data will have several clinical implications, such as providing evidence for an appropriate monitoring schedule in CHB mothers after delivery. Further analyses on predictors of such events may assist clinicians in identifying mothers who might develop severe postpartum ALT flares. The data generated from healthy mothers have the potential to identify the patterns of ALT changes during pregnancy and postpartum, so we can gain a better understanding of the normal range of ALT in this subpopulation.

**Trial Registration Number at the Chinese Clinical Trial Registry:**

ChiCTR2200061130.

## Background

Hepatitis B virus (HBV) infection is a serious threat to public health globally with a prevalence rate of up to 7% in Asia and Africa [1]. Chronic hepatitis B infection affects approximately 800 million individuals in China. Among them, approximately one million people die from cirrhosis or hepatitis B-associated liver cancer annually [1]. In the area with a high prevalence of hepatitis B, MTCT contributes largely to the new cases of HBV chronic infection [2]. The World Health Organization (WHO) has set a goal to eliminate HBV infection by 2030. Thus, the prevention of MTCT and the management of mothers with hepatitis B are critical steps to achieve the goal set by the WHO. It is estimated that the prevalence rate of hepatitis B infection in childbearing-age women in China is 6.61% [3]. Current practice guidelines [4–6] recommend that mothers with HBV DNA levels over 200,000 IU/mL should receive antiviral treatment during pregnancy to reduce the risk of MTCT. However, detailed guidance is lacking on the postpartum management of mothers with chronic hepatitis B. The practice patterns are inconsistent across different clinical settings in terms of when to stop the antiviral therapy and how to monitor mothers after delivery due to lacking high-quality evidence.

In young mothers with hepatitis B infection and high viral load without significant hepatitis, postpartum disease activation or hepatitis flare has been reported in the range of 15-35% [7, 8]. In a multicenter and prospective randomized controlled trial (RCT), Pan et al. observed that postpartum ALT elevation occurs in both mothers who were treated with tenofovir disoproxil fumarate (TDF) or received no therapy during pregnancy (45% vs. 30%) [9]. Another prospective study also showed that 36.3% of pregnant women who received antiviral therapy during pregnancy had postpartum hepatitis flares. Some of the ALT flares might further progress to acute exacerbation of hepatitis or even hepatic decompensation without timely antiviral treatment [7, 8]. In mothers without antiviral treatment during pregnancy, a large retrospective cohort study with 4236 hepatitis B mothers in China found that the postpartum ALT elevation rate was 28.27% [9]. Several independent predictors for postpartum ALT elevations were found in this subpopulation, which included high viral load or elevated transaminase during pregnancy. The peak period of ALT flares occurred between postpartum weeks 4–6 and weeks 9–12, with a bimodal distribution [9]. However, the study noted several limitations, including the lack of data on maternal virological features such as genotype, basal core promoter, pre-core mutation, and pre-S mutant. In addition, the postpartum follow-ups were within 12 weeks in most mothers. In chronic hepatitis B patients at the immune tolerance phase, basal Core Promoters (BCP) and pre-core (PC) mutations are common in HBeAg-positive patients (up to 60%) and have been linked to immune activation [10, 11]. Recently, a study by Luo et al. [12] suggests that the mutations of PC and BCP in HBeAg-positive pregnant women during antepartum could predict spontaneous HBeAg seroconversion after delivery. However, the postpartum elevation of ALT or hepatitis B flares has not been clearly described and analyzed.

Because of the inconsistent data and the lack of details, the current International Association Guidelines have not provided clear recommendations and a risk-stratified monitoring schedule. Furthermore, data are lacking on the definition of normal ALT in the postpartum period in mothers with chronic hepatitis B. Lastly, the clinical features and ALT flare patterns have not been well defined in HBeAg-positive mothers versus HBeAg-negative mothers. Furthermore, fewer data are available to address differences regarding the normal range of ALT between pregnant mothers and the general population. In a cross-sectional study by Kushner T et al. [13], the prevalence of elevated ALT in pregnancy was assessed. The authors suggested that abnormal ALT in pregnant mothers should be defined as ALT ≥ 25 U/L. However, Lee SM et al. [14] proposed a different normal range of ALT by assessing the 95 percentile ALT value in mothers in the first trimester. The authors defined the upper limit of normal as 30 U/L. The study also showed that elevated ALT to levels above 30 U/L in early pregnancy was associated with the risk of subsequent development of gestational diabetes and preeclampsia in late pregnancy. Because of the controversy on the definition of normal ALT, we believe that our study on ALT levels in healthy mothers not only helps to clarify the appropriate reference range for ALT but also takes an important step to further define ALT flares in mothers with CHB. We hypothesize that the frequency and severity of postpartum ALT flares differ between chronic hepatitis B mothers and healthy mothers. Furthermore, the patterns of postpartum ALT flares may also be influenced by the HBeAg status or antiviral therapy during pregnancy. Therefore, we will conduct a prospective cohort study and assess the incidence of postpartum ALT flares in mothers with chronic hepatitis B with subgroup analysis based on HBeAg status. In addition, we evaluate the normal range of postpartum ALT in healthy mothers without hepatitis B. In mothers with hepatitis B, the independent risk factors for predicting postpartum ALT flares are also investigated. Our data will serve as a piece of evidence to assist healthcare providers in the better management of mothers with hepatitis B.

## Methods

### Research setting

This single-center cohort study will enroll mothers with chronic hepatitis B and healthy mothers in a university medical center in China. Patients are eligible for the study after the screening with the following inclusion and exclusion criteria:

#### Inclusion criteria

1. HBsAg (+) group: pregnant women aged 20–35 who are HBsAg-positive for more than 6 months, singleton pregnancy, gestational age between 12 and 24 weeks, HBV DNA > 2000 IU/mL, ALT normal for 2 consecutive times (at least ≥ 30 days apart), Liver stiffness measurement (LSM) < 7.4Kpa and controlled attenuation parameters (CAP) < 259 dB/m;
2. Healthy pregnant women group: During the same period, pregnant women aged 20–35 with negative HBsAg and HBcAb who are treated in the Fifth Affiliated Hospital of Guangzhou Medical University, singleton pregnancy, and have two consecutive normal ALT (at least 30 days apart), LSM < 7.4Kpa and CAP < 259 dB/m. (Basically matched with the pregnant mothers in the HBsAg (+) group in terms of age, body Mass Index (BMI), and parity)

#### Exclusion criteria

1. Co-infection of the human immunodeficiency virus (HIV), hepatitis D virus (HDV), hepatitis C virus (HCV), syphilis;
2. Previous pregnancies with a history of miscarriage or congenital malformation;
3. Treatment experience (unless antiviral drugs had been used in previous pregnancies to prevent MTCT and were discontinued more than 6 months before the current pregnancy);
4. History of renal insufficiency; evidence of liver cancer or decompensation, estimated creatinine clearance rate (CLCr) < 80ml/L;
5. Clinical symptoms of threatened abortion; ultrasonic evidence of fetal malformation;
6. Co-committed use of nephrotoxic drugs, steroids, cytotoxic drugs, non-steroidal anti-inflammatory drugs, or immunomodulators; recipients of solid organ or bone marrow transplants; or diseases including severe renal, cardiovascular, pulmonary, or nervous system at the discretion of researchers;
7. Fetal biological father is infected with chronic hepatitis B;
8. ALT increased during this pregnancy before admission;
9. Thyroid dysfunction;
10. Chronic liver disease except for hepatitis B;
11. Previous pregnancy complications, such as intrahepatic cholestasis of pregnancy (ICP), gestational hypertension, gestational diabetes, and proteinuria in pregnancy.

Subjects meeting any exclusion criteria will be excluded from participating in this study. Written informed consent must be obtained from all participating pregnant women before any screening activities are initiated.

### Research scheme

**Fig. 1:**
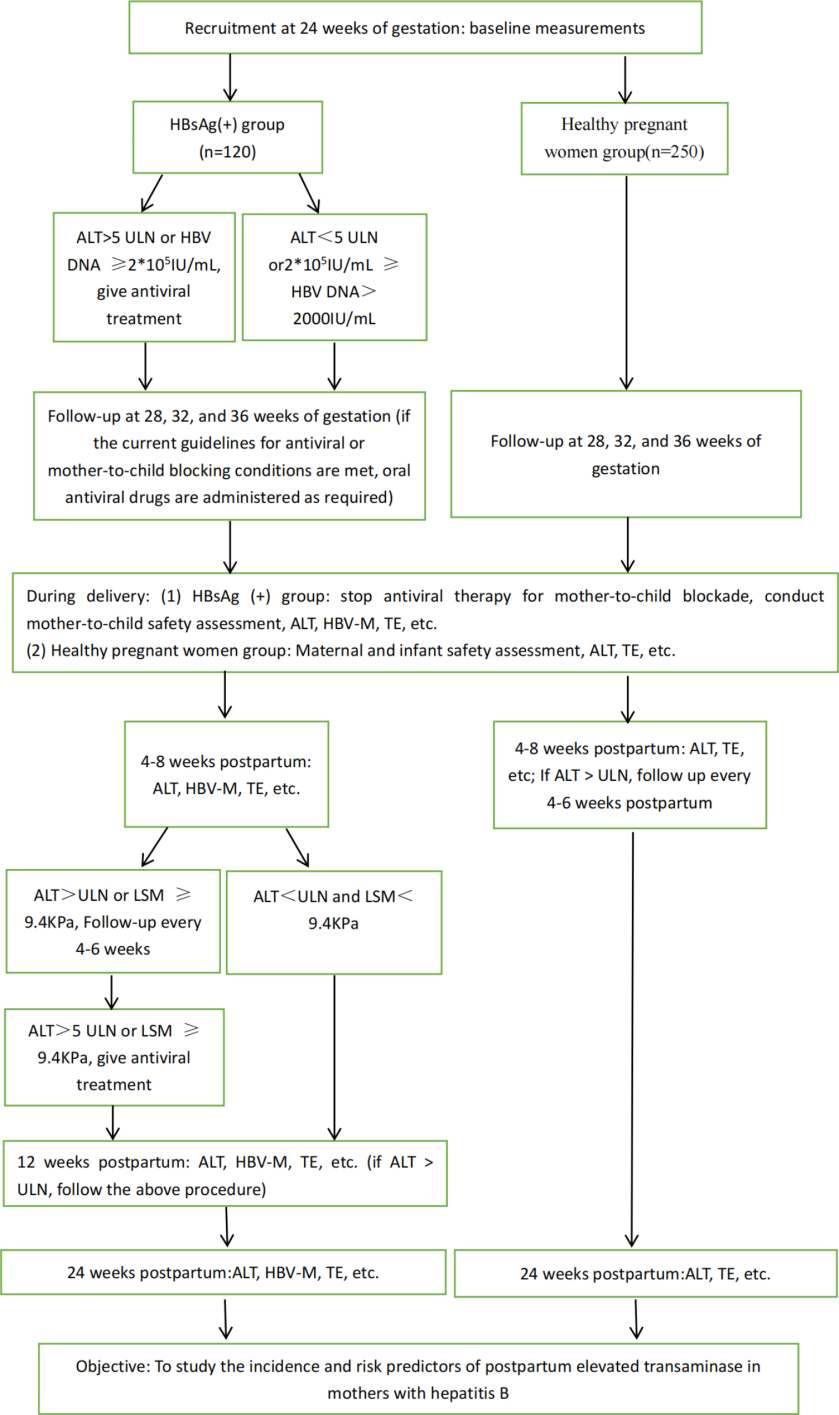
Patient Enrollment and Assessment

This study will be a single-center, prospective, open-label cohort study, from 12 to 24 weeks of pregnancy to postpartum week 24. Eligible pregnant women who are registered in the Fifth Affiliated Hospital of Guangzhou Medical University for delivery are enrolled at the 20–24 weeks of gestation and assigned into the HBsAg (+) group and healthy pregnant women group based on their HBsAg status. Patients are monitored every 4 weeks until delivery and then every 6, 12, and 24 weeks postpartum (Fig. 1). The recruitment of patients for this study began on June 1, 2022, and is expected to be completed on June 1, 2023.

### Recruitment

Subjects will be recruited through the Department of Infectious Diseases and Obstetrics Clinic of the Fifth Affiliated Hospital of Guangzhou Medical University. Recruitment began in June 2022. Based on the published literature [7, 9, 15] the frequency of elevation ALT in HBV-infected pregnant women during the postpartum period is estimated at the range of 11.6–30.6%. We calculate the sample size for the current study (presume p = 26%, q = 10%, type I error α = 0.05, test efficiency 1-β = 0.9) in the HBsAg (+) group and set the number of patients to approximately 120. In the healthy mother group, 240 subjects are planned to be enrolled (HBV mothers: healthy mothers at the 1:2 ratio). The total of subjects in the study will be 360. We estimate about 5-10% of subjects may withdraw from the study. The total number of patients who have completed data at the end of the study will be 108 in the HBsAg (+) group and 216 in the healthy pregnant women group, respectively. The sample size will provide enough to observe the adverse events with an incidence of 3% or greater.

### Trial intervention

Although our study is observational, due to the compliance with the practice guidelines recommended by the national professional association, mothers with high levels of HBV viremia will receive antiviral treatment during pregnancy and all infants will receive HBIG and HBV vaccines. These medical products are independently prescribed for participants in the study and are part of a standard care treatment strategy, which is not determined in advance by our research protocol. These treatments are applied based on clinical practice. However, we will assess the safety of mothers and newborns who receive the intervention. There will not be trial-specific intervention implemented in the study.

### 1) Antiviral therapy

In the HBsAg (+) group, antiviral therapy will be initiated if HBV-DNA levels are greater than 200,000 IU/mL at gestational weeks 24–28 until delivery. During the antiviral treatment, subjects who confirm CLCr < 50ml/min will receive the adjusted dose of the study drug based on the renal function and be monitored closely at 4-week intervals.

### 2) Infant immunization

All newborns from HBsAg (+) pregnant women will receive hepatitis B immunoglobulin (HBIG,100IU) with the first dose of the hepatitis B vaccine (10 µ g) within 12 h after birth. The second and third injections of the hepatitis B vaccine will be administrated intramuscularly at the age of 1 month and 6 months, respectively. For all healthy pregnant women, their newborns will receive the same hepatitis B immunoprophylaxis regimen and schedule without the birth dose of HBIG for infants.

.

### Laboratory assessments

Laboratory tests will include the following: liver function and biochemistry, HBV serological testing, HBV DNA, and HBV genotype. The imaging tests will include transient elastography (TE) and sonogram. We also assess the HBV virus mutation including pre-S, BCP, and PC.

### Study endpoints

The primary endpoints are the proportion of HBsAg (+) pregnant women with elevated ALT within 24 weeks postpartum and the frequency of persistently elevated ALT for more than 12 weeks in HBsAg (+) pregnant women. Secondary endpoints include the proportion, peak, and period of ALT elevation in healthy pregnant women within 24 weeks postpartum; and the comparison of the secondary endpoints between the HBsAg (+) pregnant women group and healthy pregnant women group.

### Data collection

The following variables at the baseline will be collected:

1. Age.
2. BMI.
3. History of hepatitis B.
4. Reproductive history.
5. Complication.
6. Drugs that have previously been taken.
7. Baseline laboratory data: including total bilirubin, ALT, aspartate transaminase (AST), and TE. In addition, HBsAg, HBsAb, HBeAg, HBeAb, HBcAb, HBV DNA, genotype, pre-S, BCP, and PC were detected in HBsAg (+) group.

The subsequent data collection during follow-up visits is presented in Table 1.

**Table 1:** Patient follow-up schedule and data collection

Visit(week)	12–20	Baseline(20–24)	28	32	36	delivery	4–8	12	24
Confirm eligibility	√	√							
Seek informed consent	√								
Medication review	√								
Medical history collection	√	√	√	√	√	√	√	√	√
Physical examination	√	√	√	√	√	√	√	√	√
Fetal ultrasound results	√								
Hematology laboratory tests	√	√	√	√	√	√	√	√	√
TE tests	√	√				√	√	√	√
Detection of HBV Virus Genetic Characteristics		√				√			√
Adverse Event Evaluation		√	√	√	√	√			
Weight management, dietary guidance	√								
Fetal physical examination						√			
Combined immunization						√			

Blood tests include a chemistry profile and HBV serological testing (HBsAg, HBsAb, HBeAg, HBeAb, and HBcAb) along with the HBV DNA levels. The detection of HBV viral genetic mutations includes pre-S, BCP, and PC.

### Data management

Patient-derived metadata will be de-identified and uploaded to a research database with privacy protection. Once uploaded it will not be possible to alter the data. Only the PI and research associate of the current project will have access to data.

### Patient and public involvement

Patients or the public are not involved in the current study’s design, conduct, reporting, or dissemination plans.

### Statistical methods

The statistical analyses will be performed with SPSS 23.0 version (IBM, New York, USA) statistical analysis software. Demographic and baseline measurements will be summarized using standard descriptive methods and stratified by the study groups. Baseline data for mothers will include a summary of previous gravidity, log10 HBV DNA levels, and ALT levels. The baseline data for infants will include a summary of body weight, height, head circumference, gestational age, delivery mode (cesarean section vs. virginal delivery), Apgar score, HBsAg positivity at birth, and HBeAg positivity at birth. The aforementioned baseline variables between the two groups of ALT elevation and ALT normal after delivery. The quantitative data will be summarized with a mean (SD) or median (range), and the differences between groups will be compared by a student’s t-test. The categorical data will be presented with counts or percentages and compared by the chi-square or non-parametric tests. Further analysis of predictors for ALT flares will be conducted by multivariate analysis in HBsAg positive pregnant and the area under the ROC curve will be used to predict ALT rise. A mixed linear model will be established by repeated measurement and missing value analysis to analyze the changing trend of ALT after delivery. A nested case-control study will be used to analyze the baseline risk factors for elevated ALT (further subgroup analysis of mild and severe elevated ALT). We consider the results to be statistically significant differences when the P value is < 0.05.

### Research ethics approval

This research project will be conducted by following the ethical principles of the “Declaration of Helsinki”. The current protocol (protocol number: V1.2-2022MAY13) has been peer-reviewed when applying for the research grant from the “Belt and Road” Innovative Talent Exchange Project for Foreign Experts, sponsored by the Ministry of Science and Technology, China (G2022030048L). The Ethics Committee of the Fifth Affiliated Hospital of Guangzhou Medical University approved the study on May 23, 2022 (approval number GYWY-Y2022-56). The study is still opening for enrollment. The results of this study will be submitted for publication in a peer-reviewed journal.

### Confidentiality

The investigators, or research team members designated by the investigators will be responsible for communicating with the subjects including the explanation of the study’s purpose, method, benefits, and risks. The research team will also explain to the subjects the right to participate in the study and their ability to withdraw from the study at any time for any reason. Patients who withdraw from the research project will receive the standard of care. The patient’s rights and the quality of care will not be affected. Fully informed patient consent will be provided. All records for patients who participate in the study will be confidential and data will be deidentified before analyzing or publishing in a peer-reviewed journal.

### Dissemination policy

The final data will be published in appropriate journals in the form of an original article.

## Discussion

The current article introduces the research background and research plan. At present, the study is in the recruitment stage. A previous study showed ALT flares occurred with higher frequency in mothers who received TDF therapy during pregnancy when compared to those without the treatment (45% vs. 30%) [9]. However, data are lacking on defining the normal range of ALT after delivery in mothers with HBV or healthy mothers during postpartum monitoring. The same study also suggested that elevated transaminase in pregnancy was an independent risk factor for predicting the recurrence of postpartum hepatitis [9]. However, viral mutation including pre-core or basal core status has not been well studied. Another study performed at the Nanjing University Medical College observed that mutations in PC and BCP in HBsAg (+) pregnant women during childbirth can predict spontaneous seroconversion of maternal HBeAg [12]. However, the frequency and predictors for postpartum ALT flare of hepatitis B in pregnant women were not explored in the aforementioned study. Furthermore, the effects of antiviral cessation after delivery on the elevation or deterioration of ALT have not been fully investigated. Therefore, prospective studies with high-quality data are urgently needed to establish a model for managing postpartum ALT abnormalities in HBV-infected mothers.

In the current study, we aim to investigate the frequency and severity of postpartum ALT elevation in mothers with chronic hepatitis. We analyze predictors of postpartum ALT elevation in hepatitis B-infected women through the assessments of clinical, virological characteristics, and genomics features. Our study will provide high-quality evidence for clinicians to identify mothers at risk of postpartum hepatitis. We also investigated the changes in postnatal ALT in mothers without hepatitis B and explored the normal range of ALT. The current study has some limitations, which include a non-randomized design and a lack of long-term follow-up. In conclusion, this study aims to assess the data on HBV-infected mothers and compare them with data collected from healthy mothers. The analysis will provide evidence for formulating standardized follow-up plans for hepatitis B pregnant women, which may improve the care of hepatitis B pregnant mothers.

### Strengths and limitations of the study

- This study is to investigate the frequency and severity of postpartum ALT flares in chronic hepatitis mothers, which have been reported with inconsistency in published studies.
- We also investigate the postpartum ALT changes in mothers without hepatitis B and explore the normal range of ALT, which has not been clearly defined in this subpopulation.
- Our data will clarify the maternal independent risk factors during pregnancy for predicting postpartum ALT flares in mothers with chronic hepatitis B through multivariate analyses.
- The study also compares the frequency and features of postpartum ALT flares between HBeAg-positive mothers versus those with HBeAg-negative, providing data for making clinical decisions for further intervention or monitoring based on HBeAg status.
- Our study inherits the short-coming of the cohort study without randomization and the limitation of short-term patient follow-up. However, our data may provide a road map for future studies.

## Data Availability

The datasets used and/or analyzed during the current study are available from the corresponding author upon reasonable request.

## Abbreviations

ALT: Alanine aminotransferase
AST: Aspartate transaminase
BCP: Basal Core Promoters
BMI: Body Mass Index
CAP: Controlled attenuation parameters
CLCr: Creatinine clearance rate
HBV: Hepatitis B virus
HBIG: Hepatitis B immunoglobulin
HCV: Hepatitis C virus
HDV: Hepatitis D virus
HIV: Human Immunodeficiency virus
ICP: Intrahepatic cholestasis of pregnancy
LSM: Liver stiffness measurement
MTCT: Mother-to-child transmission
PC: Precore
RCT: Randomized controlled trial
TDF: Tenofovir disoproxil fumarate
TE: Transient elastography
WHO: The World Health Organization
